# New Horizons: the value of UK Biobank to research on endocrine and metabolic disorders

**DOI:** 10.1210/clinem/dgac407

**Published:** 2022-07-06

**Authors:** Jelena Bešević, Ben Lacey, Megan Conroy, Wemimo Omiyale, Qi Feng, Rory Collins, Naomi Allen

**Affiliations:** Oxford Population Health (Nuffield Department of Population Health), University of Oxford; Oxford Population Health (Nuffield Department of Population Health), University of Oxford; Oxford Population Health (Nuffield Department of Population Health), University of Oxford; Oxford Population Health (Nuffield Department of Population Health), University of Oxford; Oxford Population Health (Nuffield Department of Population Health), University of Oxford; Oxford Population Health (Nuffield Department of Population Health), University of Oxford; UK Biobank, Stockport, Greater Manchester, UK; Oxford Population Health (Nuffield Department of Population Health), University of Oxford; UK Biobank, Stockport, Greater Manchester, UK

**Keywords:** endocrinology, metabolic disorders, review, UK Biobank, cohort

## Abstract

UK Biobank is an intensively characterized prospective study of 500 000 men and women, aged 40 to 69 years when recruited, between 2006 and 2010, from the general population of the United Kingdom. Established as an open-access resource for researchers worldwide to perform health research that is in the public interest, UK Biobank has collected (and continues to collect) a vast amount of data on genetic, physiological, lifestyle, and environmental factors, with prolonged follow-up of heath conditions through linkage to administrative electronic health records. The study has already demonstrated its unique value in enabling research into the determinants of common endocrine and metabolic diseases. The importance of UK Biobank, heralded as a flagship project for UK health research, will only increase over time as the number of incident disease events accrue, and the study is enhanced with additional data from blood assays (such as whole-genome sequencing, metabolomics, and proteomics), wearable technologies (including physical activity and cardiac monitors), and body imaging (magnetic resonance imaging and dual-energy X-ray absorptiometry). This unique research resource is likely to transform our understanding of the causes, diagnosis, and treatment of many endocrine and metabolic disorders.

Substantial progress has been made over the past few decades in understanding the causes of many endocrine and metabolic diseases (1-6). This includes the discovery of a large number of genetic variants that increase risk of common conditions, such as diabetes, obesity, and osteoporosis, and a greater understanding of the relevance of lifestyle and environmental factors to the onset and progression of these disease (5-9). The establishment of large-scale, well-characterized, blood-based prospective studies in a range of settings has been instrumental in facilitating this research, and such studies are likely to play an increasingly important role in the coming years (10).

The Medical Research Council (UK) and Wellcome played a key role in the decision to establish UK Biobank—a large-scale population-based cohort study with detailed genomic, phenotypic, and longitudinal health information (Figure 1) (11). From its inception, the resource was designed within an open science framework to enable researchers worldwide to perform health-related research in the public interest (11, 12). UK Biobank is now uniquely placed to enable researchers to make robust scientific discoveries into the determinants of a wide range of conditions, including endocrine and metabolic diseases.

**Figure 1.**
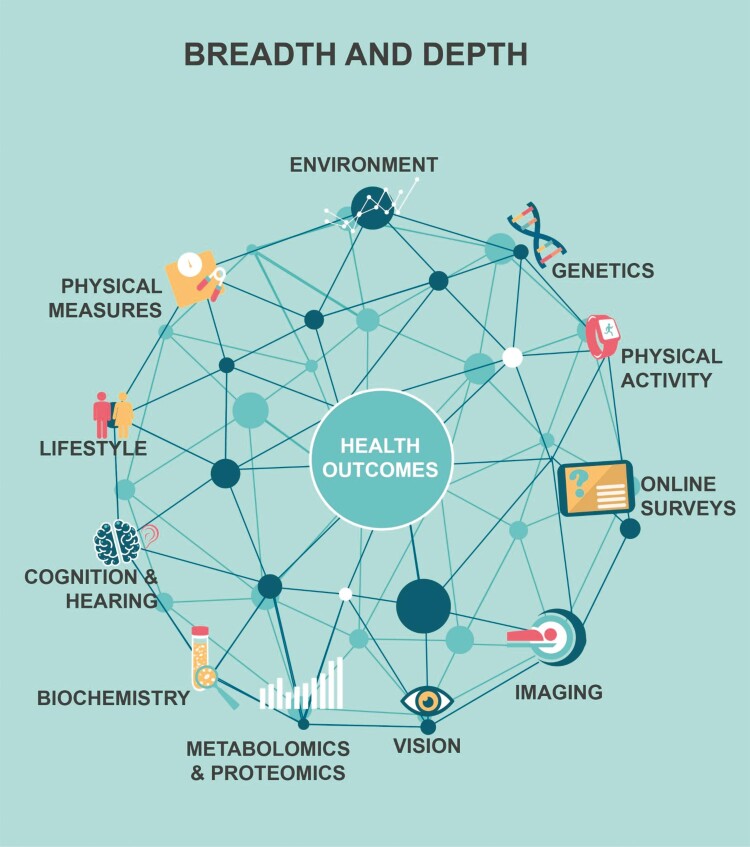
Breadth and depth of data in UK Biobank.

## UK Biobank

### Recruitment and Data Collection

Between 2006 and 2010, 500 000 individuals aged 40 to 69 years were recruited into the study from the general population of the United Kingdom. Individuals were invited to participate if they were registered with the National Health Service and lived within ~25 miles of one of the 22 assessment centers. At the recruitment visit, participants completed a wide range of assessments. Self-administered electronic questionnaires were used to collect information on sociodemographic factors, family history, early life exposures, psychosocial factors, lifestyle, health status, and environmental exposures. This was supplemented with an interviewer-administered questionnaire that obtained more detailed responses to some questions, such as on medical history. A range of physical measures were taken, including blood pressure, heart rate, grip strength, spirometry, and anthropometry (standing and sitting height; weight and bio-impedance; hip and waist circumference). Blood, urine, and saliva samples were also collected. A proportion of the cohort then underwent a hearing test, an eye examination (including refractive index, intraocular pressure, retinal photography, and optical coherence tomography), a cardiorespiratory fitness test, and a calcaneal ultrasound for bone density. Linkage to environmental factors, such as air and noise pollution, and metrics of the natural and built environment have also been derived from participants’ location of residence at the time of recruitment. Further details of the baseline data collected from UK Biobank participants are included in Table 1. Of the 500 000 participants recruited, 54% are female, 94% are of White ethnic background, and 40% reported being educated to degree level (13). Further details of the distribution of UK Biobank participants can be found on the UK Biobank data showcase (https://biobank.ndph.ox.ac.uk/showcase/search.cgi)

**Table 1.** Baseline and resurvey data in UK Biobank

Data type	Details	Number of participants	Date of collection	Date first available
Baseline questionnaire	Sociodemographic factors, family history, psychosocial factors, local environment, lifestyle, health status, medical history, cognitive function	Whole cohort (baseline)	2006-2010	2012
		20 000 (first resurvey)	2012-2013	2013
		100 000 target (imaging visit)	2014-	2014^*a*^
		60 000 target (repeat imaging)	2019-	2019
Baseline physical measures	Blood pressure and heart rate, hand grip strength, anthropometry (including bio-impedance), spirometry, heel bone density, arterial stiffness, hearing, eye examination, ECG (at rest and during activity)	Whole cohort (baseline)	2006-2010	2012
		20 000 (first resurvey)	2012-2013	2013
		100 000 target (imaging visit)	2014-	2014^*a*^
		60 000 target (repeat imaging)	2019-	2019
Web-based questionnaires	24-h diet recall (4 occasions)	210 000	2011	2012
	Cognitive function	121 000	2014	2015
	Occupational history	121 500	2015	2015
	Mental health	158 000	2017	2017
	Digestive health	176 000	2017	2018
	Food preferences	174 000	2019	2020
	Pain	169 000	2019	2021
Physical activity monitor	Accelerometer data on duration and intensity of physical activity	100 000	2013-2016	2016
		2500 (repeat measurements)	2018	2019
Imaging assessment	Abdominal, brain, and heart MRI; full-body DEXA; carotid ultrasound; ECG	100 000 target (imaging visit)	2014-	2014^*a*^
		60 000 target (repeat imaging)	2019-	2019
Cardiac monitor	14 days continual ECG to assess atrial fibrillation	36 000 target	Pending	Pending

Abbreviations: DEXA = dual-energy X-ray absorptiometry; ECG, electrocardiogram; MRI, magnetic resonance imaging.

^*a*^Data currently available for 50 000 participants. Detailed information on the data available in UK Biobank can be found on the UK Biobank data showcase: https://biobank.ndph.ox.ac.uk/showcase/.

### Outcome Ascertainment

UK Biobank participants consented to have their health followed over time by linkage to routine health-related records. To date, electronic linkage has been established to national death and cancer registrations, hospital inpatient admissions, and, for a subset of the cohort, primary care records (Table 2). These datasets provide comprehensive coverage and allow the identification of health events among participants both before and after recruitment. Primary care data are particularly useful adjuncts to the other linkages because they include conditions that are unlikely to result in hospitalization but nonetheless have high morbidity, including common endocrine disorders, such as diabetes, polycystic ovarian syndrome, and thyroid disease. Owing to the complexity and disparate nature of health care records across the United Kingdom (for example, different data providers often use different coding classifications to record diseases and procedures), algorithms have been developed to enhance diagnostic validity of selected conditions (including diabetes (14)). Summary variables are available to investigators of the first occurrence of a disease identified from any source (ie, death, hospitalization, primary care, self-report at recruitment), mapped to the International Classification of Diseases, Tenth Revision.

**Table 2.** Health record linkage data in UK Biobank

Data type	Details	Number of participants	Date of collection	Date first available
Death registrations	ICD-coded cause-specific mortality	Whole cohort	2006-	2012-
Cancer registrations	ICD-coded cancer diagnoses	Whole cohort	England 1971- Scotland 1957- Wales 1971-	2012-
Hospital admissions	ICD-coded diagnoses, and OPCS-coded procedures, from hospital inpatient records, including critical care	Whole cohort	England 1997- Scotland 1977- Wales 1999-	2012-
Primary care	Includes Read-coded data on diagnoses, prescriptions, and referrals	230 000	England 1938- Scotland 1939- Wales 1948-	2019-
Primary care (COVID-19 research only)	Includes Read-coded data on diagnoses, prescriptions, and referrals	Whole cohort	England 1938- Scotland 1939- Wales 1948-	2020- Pending Pending
SARS-CoV-2 antigen tests	Data on test result and date, and laboratory	Whole cohort	2020-	2020-

Abbreviations: ICD, International Classification of Diseases; OPCS, Office of Population Censuses and Surveys Classification of Interventions and Procedures.

### Enhancements to UK Biobank

UK Biobank continues to be enhanced through additional data collection from participants and from large-scale sample assays. A resurvey of ~20 000 participants, which included a repeat of the baseline assessment, was conducted between 2012 and 2013, primarily to enable researchers to quantify and adjust for regression dilution bias; the bias that results from the inaccuracy (because of measurement error or biological variation) with which a single measurement of an exposure at baseline characterizes long-term average (or “usual”) levels (15).

A multimodal imaging substudy is also under way in up to 100 000 participants, with 50 000 participants already having attended 1 of the 4 dedicated centers across the United Kingdom. The assessment includes a repeat of the baseline survey, including sample collection, as well as magnetic resonance imaging of the body (with detailed scans of the brain and heart), a whole-body dual-energy X-ray absorptiometry scan, a carotid ultrasound, and a 12-lead electrocardiogram (ECG) (16). This is the most ambitious study in the world to perform population-based, multimodal imaging, and its sheer scale has necessitated the development of semiautomated methods for deriving imaging phenotypes, which are then made available to all researchers (16). As more researchers use these data, the number and type of imaging-derived variables related to the structure and function of the brain, heart, kidney, and other organs, is increasing (17-21).

Further, imaging will be repeated in up to 60 000 participants to allow a deeper understanding of the relationship between changes in the structure of major organs and the causes, and consequences, of disease. UK Biobank has already performed a repeat imaging assessment of 2000 participants (half of whom had evidence of previous infection with SARS-CoV-2, and half of whom did not) to support research into the potential long-term effects of SARS-CoV-2 infection on internal organs. This is the only study in the world with both pre- and postinfection imaging data, allowing robust research into the effect of the virus on changes in physiology.

Seven-day physical activity monitoring data using wearable accelerometers were collected on a subset of 100 000 participants between 2013 and 2016. Activity monitoring was then repeated in a subset of 2500 participants a few years later, on 4 occasions over the course of 16 months, to assess seasonal variation. Derived variables related to the intensity and duration of physical activity have subsequently been made available to researchers to facilitate a wide range of research into the potential effect of physical activity on disease (22). Such data are of particular relevance to public health because UK Biobank is one of the first large-scale epidemiological studies to provide objective measures of physical activity, which may lead to improved physical activity recommendations; guidelines are currently based on evidence from epidemiological studies using self-reported measures. UK Biobank is also using wearable technology to monitor cardiac function. A subset of participants attending imaging visits are given wrist cardiac monitors to provide 14 days’ continual ECG readout, which will be particularly valuable for work on atrial fibrillation (that is often asymptomatic) and other common cardiac arrhythmias.

Efforts are also under way to expand the range of health outcome linkages to support more precise phenotyping of health outcomes, including data from national clinical audits and screening programs (such as the national diabetic eye screening program). This will enable research into specific disease subtypes and their treatment and prognosis. Web-based questionnaires are also being used to enhance health outcomes; questionnaires are routinely administered to all UK Biobank participants with a contact email address (~330 000 participants), to identify health outcomes that are not well captured in routine health records (such as cognitive function, pain, gastrointestinal symptoms, sleep, and mental health) and to collect more detailed information on major exposures of interest (such as dietary intake, food preferences, and life events that may be of importance to mental and physical health).

UK Biobank’s policy has been, where possible, to convert the biological samples that are limited and depletable into assay data on the whole cohort. Performing sample assays for all 500 000 participants at the same time facilitates quality control and enables the data to be used for a wide range of research purposes. To date, cohort-wide data are available for hematological and other key biochemistry markers (such as glycated hemoglobin, IGF-1, and sex hormones), genome-wide genotyping (~850 000 variants directly measured and > 90 million variants imputed) (23), leukocyte telomere length (24), and whole-exome sequencing (25) (Table 3).

**Table 3.** Sample assay data in UK Biobank

Data type	Details	Number of participants	Date of collection	Date first available^*a*^
Biochemistry markers	Biomarkers assayed in plasma, serum, red blood cells, and urine samples; includes established risk factors for disease (eg, lipids for vascular disease, sex hormones for cancer), diagnostic measures (eg, HbA1c for diabetes and rheumatoid factor for arthritis), and other measures (such as liver and renal function tests)	Whole cohort (baseline)	2006-2010	2016
		20 000 (first resurvey)	2012-2013	
Infectious agents	Measurement of antibody sero-positivity status of 20 pathogens	10 000 (baseline)	2006-2010	2019
Genotyping	Genome-wide genotyping was performed using the UK BiLEVE Axiom array (~50 000 participants) and the UK Biobank Axiom Array (~450 000 participants). Approximately 850 000 variants were directly measured, with > 90 million variants imputed	Whole cohort (baseline)	2006-2010	2017
Whole exome sequencing	Whole exome sequencing measures the regions of the genome (about 2%) that are involved in coding for proteins and is particularly suitable for identifying disease-causing and/or rare genetic variants	Whole cohort (baseline)	2006-2010	2019
Whole genome sequencing	Whole genome sequencing measures the entire genome and will provide information that will complement and enhance the existing genotyping and exome data	Whole cohort (baseline)	2006-2010	2021^*b*^
Telomeres	Telomere length	Whole cohort (baseline) 20 000 (first resurvey)	2006-2010	2021
Plasma metabolites	>200 circulating metabolites (predominantly lipids) measured using NMR metabolomics platform	Whole cohort (baseline)	2006-2010	2021^*b*^
		20 000 (first resurvey)	2012-2013	
Plasma proteins	Approx. 3000 circulating proteins	57 000 (baseline)	2006-2010	Pending^*c*^

Abbreviations: HbA1c, hemoglobin A1c test; NMR, nuclear magnetic resonance.

^*a*^Date data made available to the wider research community (ie, not including the exclusive period of access offered to research groups who have funded some of the sample assays).

^*b*Data available on a subset of cohort at present (450 000 participants for whole exome sequencing; 200 000 participants for whole genome sequencing; 120 000 participants from baseline and 3000 participants from first resurvey for plasma metabolites).^

^*c*^Data expected to be available end-2022.

Whole-exome sequencing focuses on the 1% to 2% of the genome that encodes for proteins and is likely to identify genetic variants of particular relevance for drug discovery (26). Whole-genome sequencing will additionally identify variants in noncoding regions of the genome that may affect disease risk and progression. Whole-genome sequencing on all participants was completed at the end of 2021, with data on 200 000 participants made available to the wider research community in November 2021; the remaining data are expected to be made available in early 2023. UK Biobank is currently the only study in the world with such large-scale detailed genetic characterization of participants, and these data will have a major impact on our understanding of the genetic determinants of disease (27).

Cohort-wide metabolomics are also under way using nuclear magnetic resonance technology that measures > 200 circulating metabolites (including lipids, lipoproteins, fatty acids, amino acids, ketone bodies, glycolysis-related metabolites, and others). Metabolomics data are currently available for 120 000 participants and will be made available for the remainder of the cohort during the next 2 years. Assays to measure proteomics (using the O-Link platform) are also ongoing, with data on about 1500 circulating proteins for about 60 000 participants expected to be available to the researcher community by the end of 2022 (data on a further 1500 proteins is anticipated by the end of 2023). The inclusion of large-scale metabolomic and proteomic data in UK Biobank will be invaluable to further our understanding of the mechanisms through which genetic and other factors influence the development of endocrine and metabolic diseases (and the impact of these diseases on other conditions). The substantial investment from a range of commercial and academic groups to perform such large-scale genomic and other -omic assays reflects the high scientific value of these data to identify novel drug targets and treatments. UK Biobank offers an exclusive period of access (up to 9 months) to research groups who have funded some of these enhancements, before releasing the data to the wider research community.

## Endocrine and Metabolic Research in UK Biobank

UK Biobank has enormous potential to enable novel research into the genetic, physiological, lifestyle, and environmental determinants of endocrine and metabolic disorders. In particular, the release of genome-wide genotyping data on such an unprecedented scale has resulted in a rapid acceleration of our understanding of the genetic determinants of disease. In particular, these data have engendered a number of genome-wide association studies (GWAS) that have identified hundreds of novel genetic variants (single nucleotide polymorphisms) associated with an increased risk of endocrine and metabolic conditions (7, 9).

For example, a recent GWAS meta-analysis on ∼ 700 000 individuals (of whom 450 000 were from UK Biobank) identified more than 900 single nucleotide polymorphisms associated with body mass index (BMI). These findings are now being used to achieve deeper insight into the complex biology of obesity, and future studies using the imaging-derived measures of total body fat and its distribution in UK Biobank will make such associations even more precise (7). In another GWAS meta-analysis, researchers combined data from ∼ 900 000 individuals of European descent (half of whom were from UK Biobank) to find more than 200 genetic loci associated with type 2 diabetes, which has directly led to advances in potential therapeutic targets (9). GWAS studies using UK Biobank have also identified genetic variants associated with other common endocrine diseases, including thyroid disease (28) and osteoporosis (8), and with risk factors for such disease, including circulating testosterone and oestradiol levels (29).

A number of the genetic findings are directly relevant to the provision of health services. For example, the genetic data have shown that individuals who have 2 variant copies of a mutation leading to hemochromatosis (a metabolic disorder affecting iron absorption) are at substantially increased risk of developing liver cancer, rheumatoid arthritis, osteoarthritis, and diabetes (30), leading to calls for the UK National Screening Committee to introduce routine genetic testing for the mutation.

The availability of GWAS data has also facilitated the conduct of Mendelian randomization (MR) analyses in UK Biobank. MR analyses can be used to assess further the causality of associations in observational studies (31-34). MR takes advantage of the random assortment of genes from parents to offspring that occurs during gamete formation and conception to mimic the effect of a randomized controlled trial for a particular exposure (thereby reducing the potential impact for confounding and reverse causality associated with standard observational analyses). Genetic variants identified through GWAS are used to construct instrumental variables for risk factors in MR analyses (35). The method has particular promise in understanding the separate and combined effects of endocrine and metabolic factors that are often highly correlated. Recent MR studies support the causal effect of BMI on type 2 diabetes and hypothyroidism (36), serum calcium levels on coronary artery disease (37), and serum estradiol (but not testosterone) on fracture risk in men (38).

Other discoveries emerging from the genotype data include the development of polygenic risk scores. These scores combine the effects of thousands of genetic variants (each of which may have only a small effect) to improve risk prediction. Indeed, in some instances, polygenic risk scores can identify individuals with risk that is equivalent to that of monogenic mutations (39, 40). For example, a study of ovarian ageing found that women in the top 1% of the genetic risk score for premature ovarian ageing had an approximate 5-fold increased risk, equivalent to those carrying monogenic *FMR1* permutations (the most established genetic cause of premature ovarian insufficiency) (39). In another study using UK Biobank data, individuals with a high polygenic risk score for type 2 diabetes had a 9-fold increase in disease prevalence (9). This opens opportunities for improvements in medical care and population health through better prevention and treatment strategies. Studies are now ongoing to assess the use of polygenic risk scores (either on their own or combined with information on lifestyle and other biomarkers) for risk stratification purposes in routine clinical practice (41).

UK Biobank genetic data have also been used in other innovative ways. For example, to show that type 1 diabetes presents frequently throughout the first 6 decades of life, and should therefore not be considered a disease of children and young adults. The study suggests that type 1 diabetes is often misdiagnosed as type 2 diabetes in older adults because of the predominance of this type of diabetes in older adults (42). Genetic data have also been used to investigate the variation in clinical presentation of participants with established genetic conditions. For example, a study of women with X chromosome aneuploidy found that the characteristics of women with 45,X chromosomes were consistent with that of clinically recognized Turner syndrome (including short stature and primary amenorrhea), whereas those with 45,X/46,XX (mosaic Turner syndrome) were less short, had a normal reproductive lifespan and birth rate, and no reported cardiovascular complications (43). Many women with X chromosome aneuploidy undergo lifetime clinical monitoring for possible complications, but the findings of this study suggests that the clinical management of women with 45,X/46,XX mosaicism should be minimal, particularly those that are identified incidentally in middle and old age.

Beyond genotyping, researchers have started to use whole exome sequencing, which focuses on genes that encode for proteins, to identify genetic variants of particular relevance for drug discovery. For example, loss-of-function variants in the *GPR75* gene were found to be associated with a reduction in BMI, with heterozygous carriers having about 50% lower odds of obesity (44). This finding was then replicated in animal models, with knockout mice gaining much less weight than wild-type mice fed a high-fat diet, with implications for the identification of suitable drug targets. Similarly, rare nonsynonymous variants in *MAP3K15* have been associated with a reduced risk of diabetes in UK Biobank, with the finding replicated in another independent cohort. The effects were independent of BMI, and an association was detected for both type 1 and type 2 diabetes, suggesting the reduced risk is unlikely to be via the insulin resistance pathway.

In addition to understanding genetic determinants of diseases, UK Biobank has also been used by researchers to investigate other major determinants of endocrine and metabolic diseases, including osteoporosis (45, 46), thyroid disorders (28), diabetes (47), and sex hormone disorders (48). These studies have often leveraged the large size of the cohort to explore associations in important population subgroups. For example, an analysis of UK Biobank used anthropometric and bio-impedance data to assess the independent associations of different body composition measures with risk of atrial fibrillation, by sex (49). The study found that, although the association of BMI with atrial fibrillation was similar in men and women, the association with lean mass and fat mass differed by sex, generating the hypothesis of different etiological pathways in men and in women.

The availability of data on levels of sex hormones and related biomarkers (such as SHBG, testosterone, estradiol, and IGF-1) for all UK Biobank participants has enabled robust research into the determinants of such factors, and in turn, their impact on disease risk (50). For example, a range of modifiable factors, including obesity, smoking, and diet, have been found to be associated with circulating testosterone concentration (48), and higher free testosterone has been associated with an increased risk of ischemic heart disease (51) and some cancers, including melanoma and prostate cancer in men and endometrial and breast cancer in women (52).

The UK Biobank resource also contains information on a range of social factors, such as qualifications, occupation (types and work patterns), social isolation, income, and area level measures of deprivation (such as Townsend Deprivation Index and Index of Multiple Deprivation). This allows for research into social determinants of adverse health outcomes. For example, a study using UK Biobank data found night shift work was associated with higher odds of type 2 diabetes (53). Other studies have explored the association between sociodemographic factors and risk of metabolic and other diseases (48, 54). UK Biobank is one of the few large-scale studies to combine detailed information on social and biological factors, offering the opportunity to improve understanding of the mechanisms by which specific social factors affect endocrine and metabolic disease; this work is likely to be particularly relevant to addressing health inequalities.

UK Biobank’s large-scale imaging project is unique, and when combined with the genetic and other health-related data, is unprecedented in enabling health-related research. Several research groups have already generated image-derived phenotypes to facilitate the use of imaging data by the wider UK Biobank research community. This includes measures of cardiac and brain structure, size of the lung, liver, pancreas, and spleen (20) and measures of body fat and its distribution, including visceral and abdominal subcutaneous adipose tissue, muscle fat infiltration, and liver fat (19, 55). All of these imaging-derived phenotypes are increasingly being used in endocrine and metabolic research (56, 57). For example, neuroimaging has been used to investigate the role of thyroid disorders in relation to regional brain volumes, with an association found between hypothyroidism and reduced gray matter volume (56).

## Accessing UK Biobank Data

Access to UK Biobank data is available to all bona fide academic or commercial researchers anywhere in the world. Applicants from low-income and middle-income countries, and students from anywhere in the world, are able to access the data at reduced cost. Following their registration, researchers must submit an application to use the resource. Applications must outline the planned health-related research and how it is in the public interest. UK Biobank also allows applications for biological samples and for recontact of participants, both of which are reviewed stringently given the depletable nature of the samples and the duty to not over burden participants.

To accommodate the increasing scale of the resource, UK Biobank has launched a Research Analysis Platform, which is now available to all approved researchers. This online platform increases the accessibility of the data, allowing access to the data for researchers who may not have the resources to download the very large volumes of data now available. It also gives researchers ready access to high-performance computing. A grant in the form of research credits is available to student researchers, early career researchers, and researchers from low-income and middle-income countries to facilitate their research using the Research Analysis Platform.

**Figure 2.**
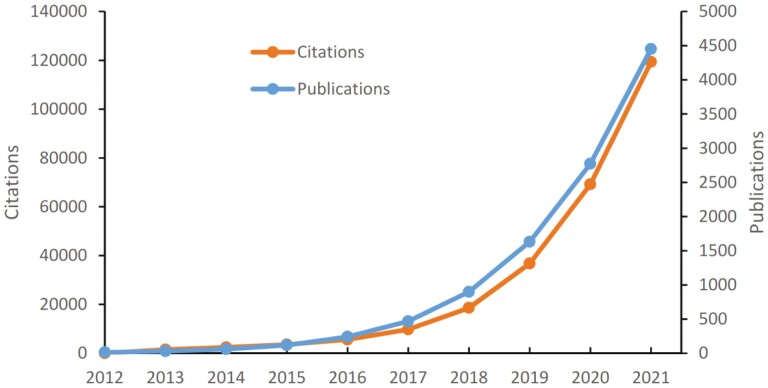
UK Biobank publications and citations 2012-2021.

By the end of 2021, UK Biobank had 27 000 registered researchers (with 80% coming from outside of the United Kingdom) and 2800 approved projects. Since 2012 (when data were first made available for research), the UK Biobank community of researchers has published more than 4500 research articles, with more than 120 000 citations (Figure 2). UK Biobank has also been referenced in > 300 patent applications.

## Conclusion

UK Biobank has become one of the most widely used biomedical research resources worldwide, enabling novel findings into the causes and consequences of endocrine and metabolic disorders. Further findings are likely to emerge in the coming years as the number of disease events accrue, and the study allows more detailed analyses on a broader range of metabolic and endocrine disorders. The resource continues to be enhanced by additional data on genetics, metabolomics, proteomics, imaging, and health outcomes, which will enable novel in-depth investigations into underlying disease mechanisms. Over the coming years, UK Biobank (together with other large blood-based cohorts from around the world) is likely to transform our understanding of the determinants and treatment of endocrine and metabolic disorders.

## Data Availability

UK Biobank is available for open access, without the need for collaboration, to any bona fide researcher who wishes to use it to conduct health-related research for the benefit of the public.

## Abbreviations

BMI: body mass index
ECG: electrocardiogram
GWAS: genome-wide association study
MR: magnetic resonance
